# Acid-base disorders after orthotopic bladder replacement: comparison of an ileal neobladder and an ileal conduit

**DOI:** 10.1080/0886022X.2017.1287733

**Published:** 2017-02-16

**Authors:** AJin Cho, Seung Min Lee, Jung Woo Noh, Don Kyoung Choi, Yongseong Lee, Sung Tae Cho, Ki Kyung Kim, Young Goo Lee, Young Ki Lee

**Affiliations:** a Division of Nephrology, Department of Internal Medicine;; b Department of Urology, Hallym Kidney Research Institute, Hallym University College of Medicine, Kangnam Sacred Heart Hospital, Seoul, Korea

**Keywords:** Conduit, ileum, metabolic acidosis, neobladder, renal function

## Abstract

**Objectives:** For many years, creation of an orthotopic neobladder after cystectomy has been popular. In the present study, we measured the extent of metabolic acidosis in patients with ileal neobladders compared with ileal conduits and defined risk factors for development of metabolic acidosis.

**Methods:** We retrospectively studied 95 patients, who underwent radical cystectomy and urinary diversion to treat invasive bladder cancer from January 2001 to December 2014 at Hallym University Kangnam Sacred Heart Hospital, through investigation of acid-base balance, serum electrolyte levels and renal function one month and one year after operation.

**Results:** One month after the operation, metabolic acidosis was found from 18 patients (31.0%) in an ileal neobladder group and from 4 (14.8%) in an ileal conduits group. One year after the operation, the numbers became 11 (22.9%) and 2 (10.0%), respectively. However, there was not a statistical difference. The blood biochemical profiles of the two groups did not differ significantly after urinary diversion. Logistic analysis revealed that lower estimated glomerular filtration rate (eGFR) was associated with metabolic acidosis at one month (odds ratio, OR = 0.94 [0.91–0.97]; *p* < 0.001) and one year (OR = 0.94 [0.92–0.97]; *P* = 0.001) after urinary diversion. In multivariate analysis, lower eGFR is a significant risk factor for metabolic acidosis at one month.

**Conclusions:** Patients with ileal neobladders and conduits are at the similar risk of metabolic acidosis. A close association between renal function and development of metabolic acidosis was observed, especially stronger in an early period after operation.

## Introduction

Radical cystectomy with urinary tract reconstruction is the standard treatment for invasive bladder cancer. After cystectomy, the most appropriate form of urinary diversion must be selected.^1^ Several such methods are available, ranging from simple incontinence with cutaneous diversion (a conduit); continence with a cutaneous diversion (a pouch); and, most recently, continent urinary diversion to the intact native urethra (featuring orthotopic neobladder reconstruction).^2^ For many years, the ileal conduit was most commonly employed. Commencing in the 1980s, orthotopic neobladder urinary diversion has found increasing favor; this eliminates the need for a cutaneous stoma and employment of urostomy appliances.^2^ To date, various forms of neobladder reconstruction have been developed in attempts to maximally normalize both anatomy and function.^3^

Although neobladders improve the quality of life, both hyperchloremic metabolic acidosis and disturbances in electrolyte metabolism are frequently reported.^4^^,^^5^ The principal cause of metabolic acidosis is absorption of ammonium ion through the intestinal mucosa. The level of metabolic acidosis is considered to be associated with the type of bowel mucosa exposed to urine and the extent of such exposure.^4^^,^^6^ Patients with preexisting disease are less able to compensate for metabolic changes. Patients with poor renal function excrete renal ammonium ineffectively.^7^

Previous studies found that mild (respiration-compensated) hyperchloremic metabolic acidosis developed after continent urinary diversion in patients with normal renal function.^8^^,^^9^ Metabolic acidosis would be expected to develop more frequently in patients with orthotopic neobladders than in those with ileal conduits. Few studies have focused on the comparison of the metabolic complications arising after the two most common forms of urinary diversion, the orthotopic neobladder and the ileal conduit. Therefore, in the present study, we explored the incidence of metabolic acidosis in patients with ileal neobladders and ileal conduits and investigated risk factors for development of the condition.

## Methods

### Patients

In total, 105 patients who underwent radical cystectomy and urinary diversion to treat invasive bladder cancer from January 2001 to December 2014 in Hallym University Kangnam Sacred Heart Hospital were evaluated. The urinary diversions featured ileal neobladders or ileal conduits. No patient had any illness (such as a severe pulmonary disorder) that could cause acidosis, and no patient was taking any medication that could trigger the condition. Ten patients were excluded because either the acid-base balance (*n* = 9) had not been noted after the operation or sepsis status (*n* = 1) had been noted. Finally, 95 patients were included.

### Measurements and outcomes

Baseline characteristics noted included demographic features (age and sex), diabetes status, bladder cancer clinical stage, and laboratory variables (creatinine, sodium, potassium, and chloride levels and blood gas data). An ileal neobladder (the bladder substitute) was created using 55 cm of the ileal segment excised from a point commencing about 25 cm proximally from the ileocecal valve. This detubularized distal ileum was used to create a spherical pouch (the Studer pouch), which was then anastomosed to the urethra. To create an ileal conduit, the ureters were surgically resected from the bladder and an ureteroenteric anastomosis then constructed to drain the urine into a detached section of the ileum. The end of the ileum was next externalized through an opening (a stoma) in the abdominal wall. When a patient could physiologically tolerate a major operation and subsequently perform normal activities, creation of an ileal neobladder was preferred.

Patients were followed up at one month and one year after operations. Each follow-up featured a clinical examination; evaluation of the blood acid-base status (measurement of pH, the anion gap [AG], and bicarbonate level); determination of the electrolyte balance (measurement of sodium, potassium, and chloride levels); and assessment of renal function (measurement of urea nitrogen and creatinine levels). Urinalysis and abdominal ultrasonography were also performed to screen for urinary tract infection and hydronephrosis. Glomerular filtration rate (GFR) was estimated according to Modification of Diet in Renal Disease (MDRD) study equation = GFR (mL/min/1.73 m^2^) = 175 × (Scr) 1.154× (Age) 0.203 × (0.742 if female) × (1.212 if African-American).^10^ Metabolic acidosis was defined as a case of a bicarbonate level lower than 21 mmol/L.^11^ We examined the metabolic acidosis status one month and one year after operation.

### Statistical analysis

All data are expressed as means ± standard deviations. Between-group comparisons were performed using the *t*-test, and the *χ*^2^ or Fisher’s exact test to compare proportions. Correlations of the serum creatinine level with the pH and bicarbonate level were calculated (Pearson correlation coefficients). Multivariate logistic regression was performed to identify independent risk factors for development of metabolic acidosis. All calculations were performed with the aid of SPSS version 18.0 (SPSS Inc., Armonk, NY, USA). A *p* value of <0.05 was considered to reflect significance.

## Results

Table 1 shows the baseline demographic and biochemical parameters of all patients by the method of urinary diversion (ileal neobladders for 62 and ileal conduits for 33). Patients with ileal conduits were more at T2a and above stage than ones with ileal neobladder. (87.9% versus 62.9%, *p* = 0.01). The mean patient age was 66.2 years (range, 40–83 years) in both groups; 64.5 years (range, 40–81 years) in the ileal neobladder group and 69.5 years (range, 51–83 years) in the ileal conduit group. No significant between-group difference in any other characteristic (sex, diabetes mellitus status, or preoperative renal function status) was evident.

**Table 1.** Baseline characteristics.

	Ileal neobladder (*n* = 62)	Ileal conduit (*n* = 33)	*p* Values
Age (years)	64.5 ± 8.6	69.5 ± 8.1	0.007
Males (%)	83.9	69.7	0.11
Diabetes mellitus (%)	14.5	18.2	0.7
Clinical stage (%)	–	–	0.01
≤T1c	37.1	12.1	–
≥T2a	62.9	87.9	–
eGFR (mL/min/1.73m^2^)	80.7 ± 20	72.7 ± 23.6	0.1
Sodium (mEq/L)	140.2 ± 2.8	138.8 ± 2.8	0.03
Potassium (mEq/L)	4.2 ± 0.4	3.9 ± 0.5	0.03
Chloride (mEq/L)	103.3 ± 3.5	102.1 ± 3.8	0.13
pH	7.43 ± 0.04	7.44 ± 0.04	0.313
PaCO_2_ (mmHg)	37.1 ± 4.6	35.9 ± 4.0	0.345
HCO3^−^ (mEq/L)	25.7 ± 2.8	25.1 ± 2.3	0.375
Base excess (μmol/L)	1.63 ± 2.53	1.20 ± 2.25	0.511

Data expressed as mean ± standard deviation.

eGFR: estimated glomerular filtration rate.

Table 2 shows the blood biochemical profiles by type of urinary diversion at one month and one year after the operation. Acid-base status could be evaluated from 85 patients at one month and 68 patients at one year. The blood acid-base status was not significantly different. Although the mean serum potassium levels at one month and serum chloride levels at one year were higher in neobladder group than ileal conduits group, the levels were within the normal range. No other significant between-group difference was apparent.

**Table 2.** Difference of blood biochemical profile according to urinary diversions.

	One month			One year		
	Neobladder (*n* = 58)	Conduit (*n* = 27)	*p* Values	Neobladder (*n* = 55)	Conduit (*n* = 13)	*p* Values
Sodium (mEq/L)	137.8 ± 4.3	136.6 ± 4.6	0.2	140.0 ± 4.2	138.7 ± 3.5	0.2
Potassium (mEq/L)	4.3 ± 0.9	3.7 ± 0.6	0.003	4.4 ± 0.7	4.1 ± 0.5	0.1
Chloride (mEq/L)	102.7 ± 13.5	101 ± 5.4	0.5	104.4 ± 4.5	101.6 ± 3.8	0.01
eGFR (mL/min/1.73m^2^)	68.1 ± 23.0	70.2 ± 24.9	0.7	61.8 ± 23.3	67.2 ± 18.1	0.3
pH	7.41 ± 0.04	7.43 ± 0.09	0.08	7.24 ± 0.9	7.42 ± 0.07	0.5
HCO3^−^ (mmol/L)	23.0 ± 5.0	25.2 ± 3.9	0.05	23.0 ± 3.9	22.8 ± 2.2	0.8
PaCO_2_ (mmHg)	35.1 ± 7.7	37.3 ± 5.8	0.2	37.5 ± 8.2	36.3 ± 7.1	0.6

Data expressed as mean ± standard deviation.

eGFR: estimated glomerular filtration rate.

Postoperative metabolic acidosis was evident in 18 (31%) patients with ileal neobladders and 4 (14.8%) with ileal conduits (*p* = 0.1) at one month after urinary diversion. At one year, 68 patients were evaluated; metabolic acidosis was evident in 11 (22.9%) with ileal neobladders and 2 (10%) with ileal conduits (*p* = 0.2) (Table 3). Although metabolic acidosis rates between two groups were different, the difference was not statistically significant. Patients with a bicarbonate level lower than 15 mmol/l were treated with sodium bicarbonate if compensation was not expected because of impaired renal function. Three patients received sodium bicarbonate at one month and six patients at one year. The metabolic status was thus fully normalized except two patients.

**Table 3.** Occurrence of metabolic acidosis in both groups.

	One month			One year		
	Normal(*n* = 63)	MA(*n* = 22)	*p* Values	Normal(*n* = 55)	MA(*n* = 13)	*p* Values
Neobladder	40 (69)	18 (31)	0.1	37 (77.1)	11 (22.9)	0.2
Ileal conduit	23 (85.2)	4 (14.8)		18 (90)	2 (10)	

Data expressed as number (%).

MA: metabolic acidosis.

**Figure 1.**
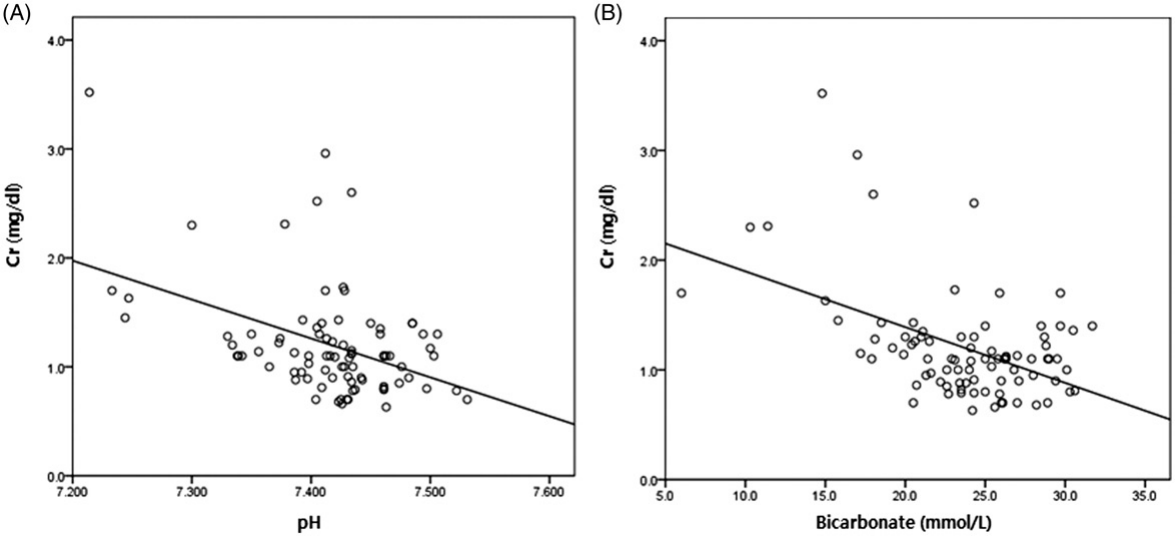
Correlation of serum creatinine and (A) pH (*r*^2^=0.19, *p* < 0.001), (B) bicarbonate (*r*^2^= 0.23, *p* < 0.001) one month after urinary diversion.

Logistic regression was performed to define independent risk factors for metabolic acidosis at one month and at one year after the operation (Table 4). In univariate analysis, lower estimated glomerular filtration rate (eGFR) was associated with occurrence of metabolic acidosis at one month (odds ratio, OR = 0.94, 95% confidence interval, CI = 0.91–0.97; *p* < 0.001) and one year (OR = 0.94, 95% CI = 0.92–0.97; *p* = 0.001). Urinary tract obstruction was also a risk factor at one year (OR = 5.67, 95% CI = 1.20–26.87; *p* = 0.03). This association was independent of age, method of urinary diversion, urinary tract infection and obstruction status (OR = 0.94; 95% CI = 0.91–0.98; *p* = 0.002) at one month. The serum creatinine level was closely correlated with the serum pH and bicarbonate level, which reflect the extent of metabolic acidosis (Figure 1).

**Table 4.** Logistic regression analysis for metabolic acidosis.

	One month		One year	
Variables	HR (95% CI)	*p* Values	HR (95% CI)	*p* Values
Univariate				
Age (per year)	1.02 (0.97–1.08)	0.4	1.10 (1.005–1.19)	0.04
Male (versus female)	1.29 (0.37–4.42)	0.7	0.56 (0.15–2.17)	0.4
Ileal neobladder (versus ileal conduit)	2.6 (0.78–8.58)	0.1	2.68 (0.54–13.37)	0.2
eGFR (per mL/min/1.73 m^2^)	0.94 (0.91–0.97)	<0.001	0.94 (0.92–0.97)	0.001
Urinary tract infection (versus no)	1.69 (0.63 –4.53)	0.3	2.07 (0.60–7.13)	0.3
Urinary tract obstruction (versus no)	1.99 (0.63–6.32)	0.3	5.67 (1.20–26.87)	0.03
Multivariate				
Age (per year)	1.0 (0.94–1.08)	0.9	1.10 (1.0–1.22)	0.1
Ileal neobladder (versus ileal conduit)	2.55 (0.62–10.51)	0.2	2.92 (0.44–19.65)	0.3
eGFR (per mL/min/1.73 m^2^)	0.94 (0.91–0.98)	0.002	0.96 (0.92–1.0)	0.06
Urinary tract infection (versus no)	1.19 (0.36–3.90)	0.8	0.83 (0.17–4.15)	0.8
Urinary tract obstruction (versus no)	1.20 (0.30–4.69)	0.8	4.53 (0.52–39.39)	0.2

eGFR: estimated glomerular filtration rate.

## Discussion

We found no significant difference between the conduit and neobladder groups in terms of serum electrolyte or blood gas levels. Metabolic acidosis was evident in 31% of patients with ileal neobladders and 14.8% of those with ileal conduits at one month of the operation, and in 22.9% and 10% of patients in these two groups at one year. Most patients with urinary diversion via a segment of intestine are at risk for metabolic disturbances. Several factors appear to exert an important influence on severity of the metabolic acidosis, including the type of urinary diversion and the portion of intestine used.^12^ The metabolic challenge posed by an ileal conduit is less than that of a reservoir because the bowel segment used is shorter and the conduit does not function as a reservoir.^13^

Many studies have reported metabolic disorders after urinary diversion. Hyperchloremic metabolic acidosis is almost always subclinical, although 10% of patients with ileal conduits were reported to have clinically significant conditions.^14^^,^^15^ In prospective series, the frequency of metabolic acidosis after continent diversion and orthotopic bladder replacement was 26%–45%.^13^^,^^16^ In the present study, 31% of patients with ileal neobladders at one month and 22.9% at one year developed metabolic acidosis after the operation. The condition was somewhat less common in those with ileal conduits, but the difference was not significant. The small subject size might have low power for validating the difference of metabolic acidosis between two methods. However, this study showed that patients with orthotopic bladder replacement using ileum did not appear to create significant acid-base changes. Although, nine patients with neobladder were administered oral sodium bicarbonate, most of them restored a normal metabolic status. No one needed hemodialysis for correction of metabolic acidosis.

Occurrence of metabolic acidosis could be evaluated from 62 patients at both times. Out of 16 patients with metabolic acidosis at one month, only four patients (33.3%) kept the acid-base status at one year. Metabolic acidosis in early period was not associated with one in late period (*p* = 0.5). Furthermore, this study demonstrated that metabolic acidosis decreases with time. This finding was comparable with previous study that patients with temporary metabolic acidosis recovered within one year after the surgery.^13^ They reported that changes in the intestinal mucosa caused by exposure to urine eventually alter the transport activity of electrolytes in the neobladder. Metabolic acidosis reportedly decreases with time, in association with significant structural changes in the ileal mucosa such as atrophy of the intestinal villi, decrease of villi, decrease of villi-to crypt ratio and mucosal fibrosis after long term exposure to urine.^17–20^ Generally, patients who have undergone urinary diversion and who have normal renal and hepatic function seem to adequately compensate for ongoing acid absorption by a neobladder. However, when patients with renal impairment undergo urinary diversion, the risk of metabolic acidosis is elevated. Patients undergoing urinary diversion following radical cystectomy suffer declines in renal function caused by age and complications of urinary reconstruction, placing them at greater risk of acid-base balance disorders.^21^^,^^22^ In the present study, we found the relationships between decreased renal function and acid-base metabolic status and especially, close association of that at one month after urinary diversion.

Ureter obstruction, recurrent infections, and urinary lithiasis can impair renal function after urinary diversion. The precise impact of urinary diversion on renal function remains unknown.^11^ Renal function falls by 15%–25% after urinary diversion.^23^ Only a few reports on renal function after cystectomy and Studer neobladder reconstruction have been published. Many studies reported functional outcomes and complications without addressing renal function. In the present study, 27% of patients developed AKI 1 month after urinary diversion, caused by ureter obstruction, urinary tract infection, medication and unknown cause. The neobladder group had a higher frequency of AKI than the conduit group, but no patient required postoperative dialysis.

Our study had several limitations. First, the work was retrospective in nature, and some patients were not subjected to follow-up blood gas analysis. Furthermore, only 65% of patients were evaluated in terms of cancer stage. Second, the follow-up duration was only one year; thus, long-term outcomes were not determined. However, metabolic acidosis after urinary diversion tens to improve within one year. Third, the small sample size might afford only a low statistical power. The significance of the associations that we found need to be confirmed in works with larger sample sizes.

In conclusion, the frequency of metabolic acidosis did not differ significantly between patients receiving ileal neobladders and ileal conduits. In addition, poor renal function and metabolic acidosis were closely associated, especially in the early period. Even severe metabolic acidosis could be relatively easily corrected by oral administration of sodium bicarbonate, suggesting that orthotopic ileal neobladders are safe, but that careful follow-up (including blood gas analysis) is required for patients with poor renal function.
